# Insights into the Seasonal Olfactory Mechanism of Geosmin in Raw Water of Huangpu River

**DOI:** 10.3390/toxics10080485

**Published:** 2022-08-19

**Authors:** Fei Luo, Hui Chen, Xiaoxin Wu, Lili Liu, Yuean Chen, Zhiping Wang

**Affiliations:** 1 China-UK Low Carbon College, Shanghai Jiao Tong University, Shanghai 200240, China; 2 School of Environmental Science and Engineering, Shanghai Jiao Tong University, Shanghai 200240, China; 3 State Environmental Protection Key Laboratory of Environmental Risk Assessment and Control on Chemical Process, East China University of Science and Technology, Shanghai 200237, China

**Keywords:** geosmin, microbial community, raw water, olfactory mechanism

## Abstract

Since the 1990s, the raw water of Huangpu River in Shanghai, China, has intermittently encountered off-flavor contamination. In this work, the concentrations of typical odor, geosmin, in raw water of Huangpu River are found to shift along with the seasons. However, microbes recognized as the producer of geosmin such as Cyanobacteria and Actinobacteria are not consistent with the shift of geosmin. Cyanobacteria blooms in summer rather than winter, whereas Actinobacteria thrives in winter. Representational difference analysis (RDA) reveals that microbes associated with blooming algae have positive co-occurrence correlations with the concentrations of geosmin and nutrients in winter, whereas those within Cyanobacteria and Planctomycete are in a positive correlation with temperature and thrive in summer. This causes the concentration of geosmin in raw water to appear to depend on the abundance of Actinobacteria rather than that of Cyanobacteria. However, combining with the synthesis and storage properties of geosmin in algae, as well as the decomposition properties of algae with Actinobacteria, geosmin might be synthesized by Cyanobacteria in summer, which is stored in cells of Cyanobacteria and released only via the decomposition of Actinobacteria in winter. This potential olfactory mechanism of geosmin is quite different from that derived from pure culture of odor producers or correlation analysis of bacteria and odors; thus, providing insights into the mechanism of practical off-flavor events.

## 1. Introduction

Since an urgent off-flavor event broke out in Pipa Lake, Japan, in the late 1960s [1], earthy odors have become the most emerging pollutants that induce consumers’ complaints around the world. Although there is still no direct evidence that odors are associated with adverse health effects, these off-flavor events produced by microbes may suggest pollution of drinking water and promote consumer distrust, resulting in large quantities of economic loss [2]. In an effort to eliminate odors from drinking water, adsorption with activated carbon and oxidation by oxidants have been investigated [3] and confirmed as costly and often unreliable treatments [4]. Therefore, many works have been carried out to prevent the production of odors and bloom of related microorganisms [5,6] and explore their production mechanism and environmental factors [7], which might propose cost-effective measures for water resource management.

In general, geosmin and 2-MIB are recognized as two typical earthy compounds, which are produced by planktonic and benthic Cyanobacteria, Actinomycetes, fungi, or *Myxobacteria* as secondary metabolites [8]. In many cases, Cyanobacteria and Actinobacteria are reported to be the major potential source of odors, and responsible for off-flavor events in aquatic environments [9] and sediment/soil [10], respectively. Therefore, water replenished by surface water is often disturbed by Actinobacteria, whereas warm water with rich nutrients favors the bloom of Cyanobacteria, and both of them might lead to off-flavor events.

Since odors are mainly produced by microbes, it is expected that concentrations of odors might be increased proportional to cell density of Cyanobacteria or Actinobacteria [11]. However, it is confirmed that most of the odors are found to be stored intracellularly in the producers rather than released out of cells immediately [12], and the outbreak of odors depends mainly on release of odors stored in cells of the producers [13]. Moreover, according to the previous pure cultures in the laboratory, the productivity of odors is heavily dependent on the living conditions of microbes, such as light, temperature, dissolved oxygen, nutrients, and growth phase [12]. Therefore, the olfactory mechanisms for most of the practical odor events are still unclear [7,8], which is important for the quality management of raw water and drinking water supplies.

Huangpu River, the main source water of Shanghai, has intermittently encountered taste and odor problems, resulting in continuous consumer complaints since the 1990s. In order to improve the quality of drinking water, ozone pre-oxidation, particle-activated carbon, and ozone-activated carbon have been employed successively to eliminate odors, resulting in a substantial increase in treatment costs as well as the secondary pollutants. In this study, besides the determination of the typical odors, geosmin, microbial communities in raw water as well as the corresponding environmental conditions, parameters of water quality are determined and analyzed via multivariate statistical analyses. A potential geosmin olfactory mechanism is proposed, which might provide theoretical and novel reference for the quality management of raw water.

## 2. Materials and Methods

### 2.1. Sample Collection and Pretreatment

In order to meet the demand of determination, 2.5 L of raw water and 50 g of coagulation sediments were collected in a regular interval from March 2016 to December 2017 from a local water supply plant, Shanghai, China. The sampling conditions were obtained in situ with a microbe sensor (Shanghai Leici Instrument Co., Shanghai, China), and the concentrations of chemical oxidation demand (COD), ammonia, and total phosphate (TP) of water samples were determined according to the standard method of EPA [14] (Table S1). Samples were immediately transported back to the laboratory at 4 °C. Each water sample (2 L) was filtered with 0.22 μm acetate fiber filter to obtain the biomass in water. Then, the filtered filters containing biomass of each sample were cut into pieces, placed in a sterilized centrifuge tube, fixed in 70% (*V*:*V*) ethanol, and stored in a −20 °C freezer before DNA extraction. In addition, the sludge samples were lyophilized under vacuum, ground, and screened with a 100 mesh sieve, which was packed in sealed vacuum and placed in a refrigerator at −20 °C until DNA extraction.

### 2.2. Determination of Geosmin

In this study, Headspace–Solid Phase Microextraction–Gas Chromatography/Mass Spectrometry (HS-SPME-GC/MS, TSQ Quantum XLS, Thermo Co., Waltham, MA, USA) was used to determine the concentration of odors (geosmin) in water samples. Firstly, 5 mL of water sample was added into a 20 mL brown glass vial pre-filled with 1.5 g of sodium chloride and warmed in a 60 °C water bath after sealing with a rubber cap, then the SPME (Supelco 57330-U, Bellefonte, PA, USA) head was inserted and extracted for 30 min with a 200× *g* magnetic stirrer. After that, the extraction head was retracted in the SPME holder and inserted into the GC-MS inlet for determining the olfactory substance concentration. The Rtx-5MS capillary column (30 m × 0.25 mm × 0.25 μm) was used for the chromatogram with high purity helium (99.999%) as the carrier gas. GC/MS analysis was performed as follows: splitless injection method, 250 °C inlet temperature, and 1.2 mL/min column flow rate; Moreover, the temperature rising mode was controlled at a rate of 15 °C/min from the initial temperature 40 °C transferring to 250 °C. Then, the transmission line was maintained for 10 min. The mass spectrometer used EI as the ion source, 70 eV ionization energy, and 230 °C ion source temperature. The scan mode was set as qualitative Full Scan (Full Scan) and quantitative SIM (Ion Detection). The chemicals and standard sample of geosmin were purchased from Sigma-Aldrich Co. (Saint Louis, MO, USA).

### 2.3. DNA Extraction and High-Throughput Sequencing

In this work, 19 samples were used to extract DNA for analysis of the microbial community, including 15 samples in summer and 4 samples in winter (Table S2). For each sample, two parallel DNA samples were extracted using a FastDNA^TM^ Spin Kit for soil (MP Biomedicals, Santa Ana, CA, USA) following the manufacturer’s instructions, and the concentration and purity of DNA was determined using a NanoDrop-1000 Spectrophotometer (Thermo Scientific, Waltham, MA, USA). Purified DNA extracts were stored frozen at −80 °C until further analysis. The DNA samples were sent to a commercial sequencing company for high-throughput sequencing, and the barcoded forward primers were used to amplify nucleotides targeting the hypervariable V3 and V4 regions. The sequencing raw reads were deposited into the NCBI Short-Reads Archive database with the accession numbers of PRJNA503301.

### 2.4. Sequence Processing

Firstly, sequencing raw data were pair-merged and filtered with a base score threshold > 30, and then processed on the Mothur platform v. 1.33 (Schloss et al., Ann Arbor, MI, USA) according to the Miseq SOP procedure to remove redundant data [15]. The non-repetitive sequences were compared with the 16S RNA gene database (RDP release 11) to get their taxon, and the distances between the sequences were calculated. The sequences with a similarity greater than 97% were assigned to the same operational taxonomic unit (OTU), and each OTU corresponded to a different 16S rRNA sequence, that is, each OTU corresponded to a different bacterial (microbial) species. The representative sequence for each OTU was chosen, the microbial diversity and the abundance of different microorganisms were then analyzed. As a result, the microbial population distribution of each sample, along with bacterial community, were determined.

### 2.5. Data Analysis

The sequencing depths of samples were calculated as coverage of a unique sequence in the reconstructed sequence, whereas microbial richness and diversities of samples were calculated using the corresponding analytical tools in RDP, represented as the Chao index and Shannon index, respectively. According to the taxon of OTUs, phyla and genera with the top 15 abundances were used to preliminarily compare the compositions of microbial communities. A Venn diagram was employed to count the number of OTUs and their represented sequences shared or unique in different samples, and illuminated the difference in microbial communities at the level of genera. An analysis of variance (ANOVA) was used to highlight OTUs with significant differences in summer and winter samples. A sorting method based on correspondence analysis of multiple regressions, redundancy analysis (RDA), was used to present the relationship between the samples and environmental factors as well as microbes. For positive network analysis, the microbial communities and environmental parameters were represented as the nodes, and the strong and significant correlations between nodes were represented as the edges. In addition, to reach the robust correlation among entities in a valid co-occurrence event, the Spearman’s coefficient was set >0.6 and statistical significance (*p*-value) was <0.01 [16]. All network analyses were performed in R platform (R-4.2.1, R core team, Vienna, Austria) using vegan, igraph, and Hmisc packages and visualized with gephi.

## 3. Results and Discussion

### 3.1. Seasonal Variation in Geosmin in Raw Water

**Figure 1:**
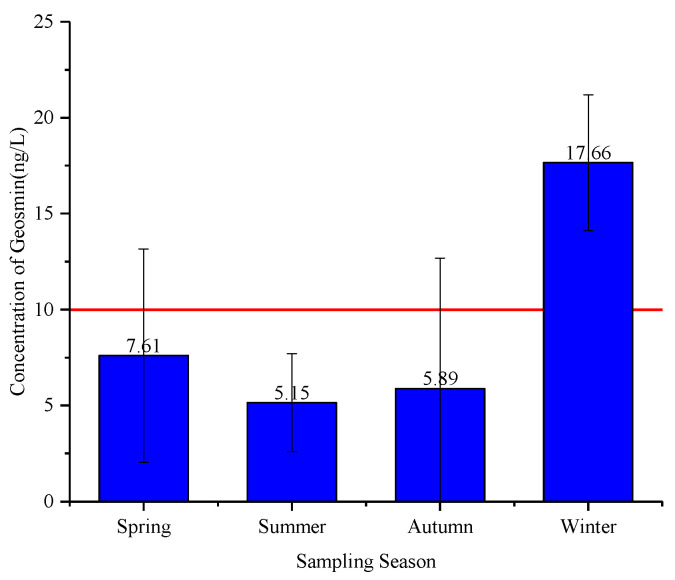
Seasonal variation in geosmin concentration in raw water of Huangpu River. The red line represented the limitation of drinking water in China.

Samples were collected in regular intervals from a local water supply plant and the concentrations of geosmin were determined using HS-SPME-GC/MS after pretreatment. According to the results, the average concentrations of geosmin in water samples range from 5.15 to 17.66 ng/L during spring to winter. The concentrations of geosmin in all summer samples are lower than the local limitation of drinking water (10 ng/L), whereas those in winter samples are higher than the limits, suggesting there might be significant differences between samples from summer and winter (Figure 1). This seasonal feature is consistent with previous research [17], which also showed higher concentration of odorous compounds in winter. The concentrations of geosmin in summer and early autumn are persistently lower than the recommended health standards (GB 5749-2022, China), which might be attributed to the inhibition of olfactory microbial communities in the raw water caused by strong light, high temperature [18,19], or the dilution of rain in the rainy season. Based on this result, samples of water and the corresponding coagulation sludge in summer and winter were collected for microbial community analysis in the following experiments, as well as the corresponding environmental parameters of samples, to explore the potential olfactory mechanism.

### 3.2. Sequencing of Microbes and Their Diversity

According to the concentrations of geosmin in raw water, 19 samples were selected to extract DNA and perform high throughput sequencing for microbial community analysis, including 15 summer samples (5 of water and 10 of coagulating sludge) and 4 winter samples (2 of water and 2 of coagulating sludge), respectively. Since coagulation is recognized as the most efficient treatment process for removal of odorous pollutants in intact cells [20] and the olfactory microbes are found to be living in attachment [21], sludge samples corresponding to raw water were used to investigate the removal efficiency of olfactory microbes in raw water, as well as the suspension characteristics of microbes.

Among of the 19 samples, the coverages of sequencing ranged from 0.96 to 0.99 (Table S2), which indicates that the sequencing depths for all samples are sufficient for the assessment of microbial communities. A total of 885,993 sequences were produced after quality control, with a minimum sequencing depth of 33,880 sequences for summer sludge (mh20) and a maximum sequencing depth of 61,724 sequences for winter water (mh29). In order to make the different samples comparable, the sequencing data of samples were normalized and classified as four categories (Water_Summer, Water_Winter, Sludge_Summer, and Sludge_Winter) before further analysis. The alpha diversity of summer samples is much higher than those of winter samples, with the mean Shannon index of 6.42 and 4.74, respectively, suggesting that the diversity of microbes in summer is much higher than those in winter. This might be ascribed to the suitable environmental and nutritional conditions in summer.

### 3.3. Seasonal Variation in Microbial Communities

**Figure 2:**
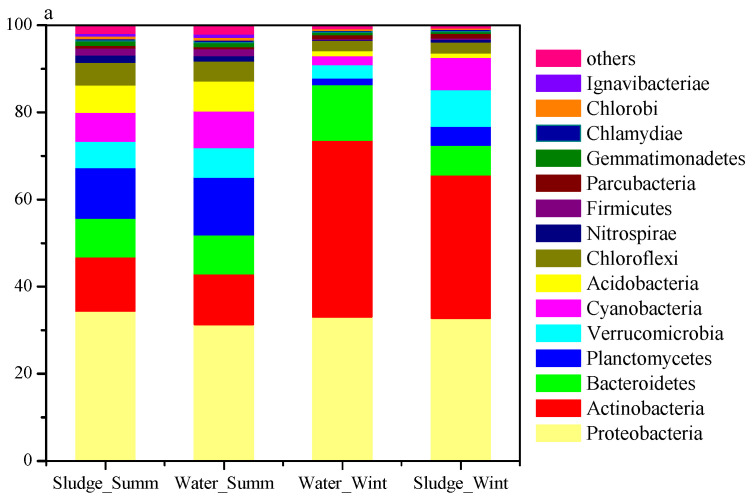

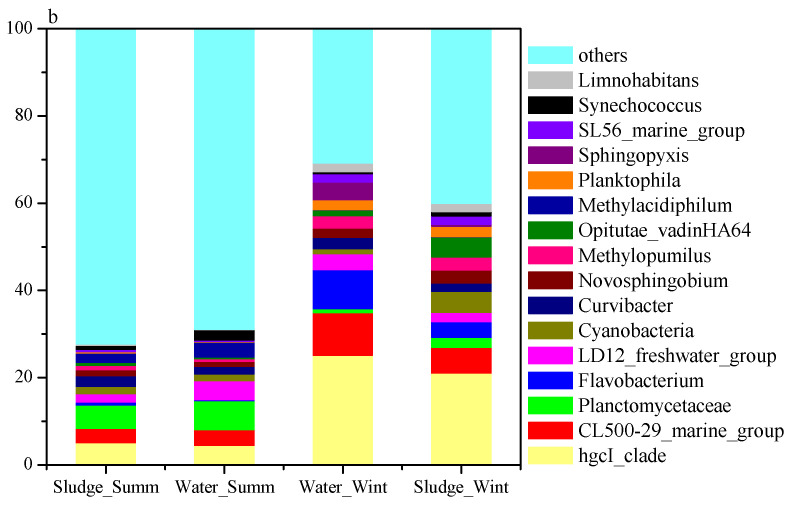
Microbial communities of raw water and the corresponding coagulation sedimentation sludge: (**a**) phylum level; (**b**) genus level.

According to the taxon of OTUs with 16S rRNA reference (RDP, Release 11), Proteobacteria, Actinobacteria, Bacteroidetes, Planctomycetes, and Cyanobacteria are ranked as the top 5 phyla, which is consistent with most surface water [22]. However, the microbial compositions of the summer samples are significantly different from those of winter samples, especially for Actinobacteria, a potential geosmin producer [1]. As the season shifted from summer to winter, the abundance of Actinobacteria in water increased from 11.4% to 40.2%, whereas the abundances of Cyanobacteria, Planctomycetes, and Verrucomicrobia are decreased in halves, respectively (Figure 2a). As the concentrations of geosmin in winter water are much higher than those in summer water, and the abundance of Cyanobacteria even decreased significantly in winter water, it seems that Cyanobacteria might not to be associated with the increase in geosmin in winter water of Huangpu River. In contrast, the abundances of Actinobacteria in winter samples are much higher than those in summer samples, implying that they are correlated with the high concentration of geosmin in winter water. However, the further classifications of OTUs at the genus level reveals that there is no well-known genus related to geosmin production, such as *Streptomyce* or *Anabaena*, even in the winter samples with high geosmin concentrations. The dominant genera in winter water are hgcI clade (*Candidatus Nanopelagicus*) and CL500-29 marine group (*Ilumatobacter*), two groups of free-living Actinobacteria abundant in many freshwater ecosystems, whereas those in summer water are hgcI clade and genera from Planctomycetaceae (Figure 2b). The hgcI clade is recognized as aerobic–heterotrophic or photoheterotrophic lifestyle [23], living mainly in epilimnetic waters and favors high water transparency and low DOC concentration, but their functional traits are still unknown. Some previous works have reported that [24] this lineage is positively correlated with the algae bloom and its growth could be accelerated with the addition of putrescine, a product of phytoplankton decomposition; thus, might be related to the decomposition of Cyanobacteria or algae.

In addition, the representative sequences of the top 50 OTUs in the four groups were used to obtain the closest related species and assign their taxon via blast on the NCBI website, and a phylogenetic tree was constructed to show their taxonomic position (Figure S1). Most of the top 50 OTUs belong to Actinobacteria, whereas *Streptomyces*, the traditionally recognized predominant genus of geosmin resource, does not exist in all samples from Huangpu River. The microbial compositions of summer water and sludge samples are almost the same, except for the Proteobacteria. However, the microbial compositions of winter water and sludge samples are significantly different. Comparing with the water samples, the percentage of Actinobacteria and Bacteroidetes in winter sludge samples decreases obviously, whereas that of Cyanobacteria and Verrucomicrobia increase greatly in sludge samples. This indicates that Cyanobacteria and Verrucomicrobia might live in attachment and could be removed more easily from raw water than Actinobacteria and Bacteroidetes via coagulation. For example, comparing with the microbial composition in winter water, the abundance of *Flavobacterium*, CL500-29 marine group, and hgcI clade in winter sludge are decreased by 59.0%, 41.3%, and 19.9%, whereas *Cyanobacteria* and *Opitutae* are increased by 309.1% and 237.6%, respectively.

### 3.4. Screening of Potential Odorous Producers

**Figure 3:**
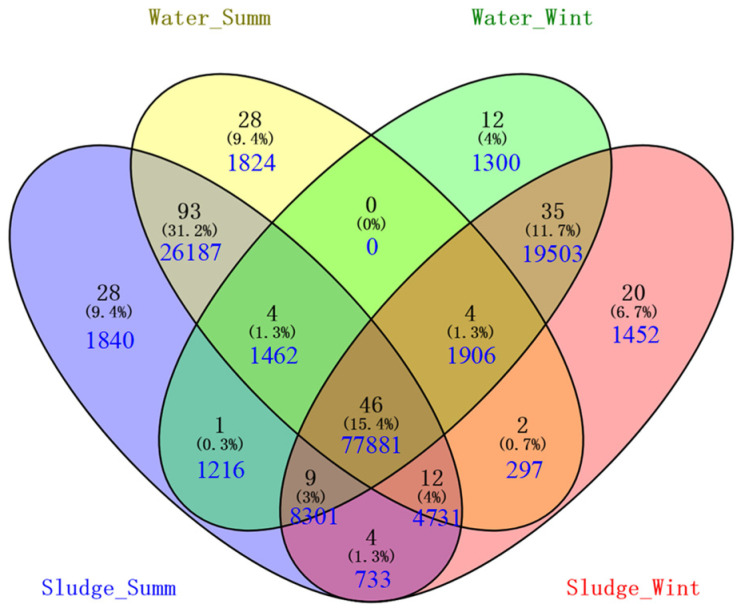
Conserved and unique OTUs in four groups (yellow, raw water in summer; green, raw water in winter; blue, sludge in summer; and red, sludge in winter).

In order to further reveal the differences in microbial compositions in the four groups, OTUs with sequences abundances greater than 1‰ within each group were selected to draw a Venn diagram (Figure 3). In theory, all microbes in the sludge samples should be included in the corresponding microbial communities of the water samples, for they are separated from water via coagulation. However, there are still many non-aqueous microbial populations presented in both seasonal sludge samples. Comparing with the sequences in water samples, sequences clustered into these extra OTUs in summer or winter sludge samples are much fewer; thus, might be ascribed to the variation in microbial population after coagulation. It should be noticed that the number of OTUs shared by all four groups is 46, accounting for only 15.4% of total selected OTUs. However, they possess 77,881 sequences in total, accounting for more than 50% sequences, indicating that the co-occurring OTUs must represent the dominant species in the four groups. The shifts in microbial community in different seasons are mainly caused by the change in relative abundance of dominant species and some of the minor species. For example, OTU3765, the shared top abundance of OTU, possesses 1165, 1179, 5418, and 4972 sequences in samples of summer water, summer sludge, winter water, and winter sludge, respectively, whereas the unique top OTU for summer and winter water, OTU3607 and OTU4008, possess only 129 and 332 sequences, respectively.

According to the significantly different concentrations of geosmin in summer and winter water, the differences in OTUs in the water samples should be inspected carefully, including the unique OTUs of winter water (12 OTUs) and the common shared OTUs of winter water and sludge (35 OTUs). For the unique OTUs of winter, OTU4008 possesses the highest abundance being about 0.22% of total sequences, which is assigned as *Flavobacterium* via Blastn on the NCBI website, a potential 2-MIB degrader decomposing 2-MIB to form 2-methylenebornane and 2-methyl-2-bornane as the products [25]. In addition, OTU4163 possesses the second highest abundance (about 0.16%), which is assigned as *Rheinheimera* via BLASTn on the NCBI website, a dominant aerobic anoxygenic phototrophic bacteria in cyanobacterial aggregates during cyanobacterial blooming that could digest *Dolichospermum crassum* completely with an inoculation dosage of 100 cells/mL [26]. For the common shared OTUs of winter water and sludge, OTU3981, OTU4014, OTU1226, OTU3711, and OTU4086 are ranked as the top 5 OTUs, with abundances of 1.50%, 1.20%, 1.12%, 1.00%, and 0.85%, respectively, which are assigned as *Sphingopyxis*, *Flavobacterium*, hgcI_clade, *Sphingomonas,* and *Verrucomicrobium* sp., respectively, via the BLASTn on the NCBI website. Among these five microorganisms, *Flavobacterium*, *Sphingopyxis*, and *Sphingomonas* have been reported as the geosmin degraders [27] and MIB degraders [28], and hgcI_clade *(Candidatus Nanopelagicus)* has been related with the decomposition of Cyanobacteria [12], while the relationship between *Verrucomicrobium* sp. and odors is still unknown. Therefore, it seems that the unique OTUs in winter water are mainly related to the Cyanobacterial aggregates or geosmin degradation rather than production.

**Figure 4:**
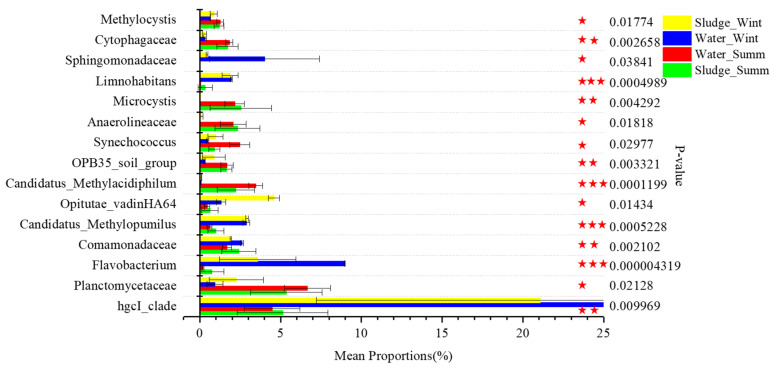
Significant differences in genera in four groups (the error bars mean standard deviation, whereas the stars mean the extent of the significant difference).

Besides the unique OTUs, those OTUs with significant differences in summer and winter water should also be noticed, which might have a significant impact on the geosmin production or release. According to the one-way ANOVA analysis, the top 50 OTUs in four groups were tested to find those OTUs with a significant difference in samples of summer and winter water. Among them, the hgcI clade, *Flavobacterium*, *Methylopumilus*, *Limnohabitans,* and *Sphingopyxis* are ranked as the top 5 genera with significant differences, and their abundances in winter water are much higher than in summer water. Based on the above analysis, *hgcI clade*, *Flavobacterium,* and *Sphingopyxis* are suggested to be connected with the blooming aggregates and decomposition of Cyanobacteria. Some researchers also find that *Methylopumilus* is mainly distributed in hypolimnetic samples and rare in surface water [29], which is of conspicuous small size and flourishes concomitantly with blooms of diatoms and/or Cyanobacteria [30]. Similarly, *Limnohabitans* is found to thrive along with the bloom of phytoplankton in spring or summer [31] and used phytoplankton-derived organic material as the key substrate for growth [32]. Therefore, all the top 5 OTUs with significant differences in samples of summer and winter are connected with the bloom of Cyanobacteria. However, Cyanobacteria represented by *Synechococcus* and *Microcystis* are significantly higher in summer than in winter; thus, the above five microbes that thrived in winter might be the consequence of blooming Cyanobacteria in summer (Figure 4).

### 3.5. Effects of Environmental Parameters on Microbial Communities

**Figure 5:**
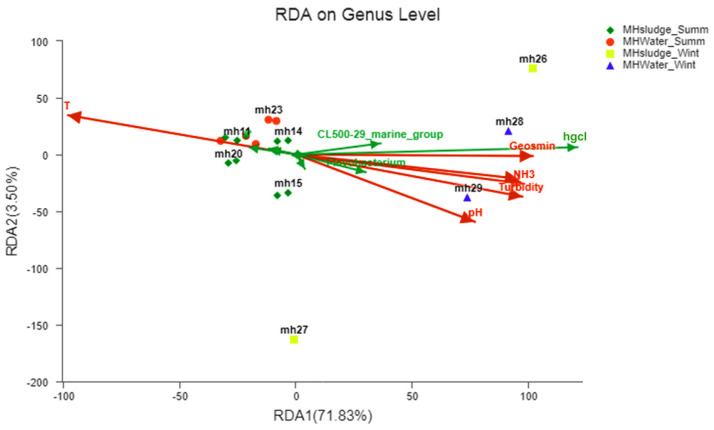
RDA analysis of the samples and environmental factors, as well as the genera (red arrow, environmental parameters; green arrow, genera).

According to the above analysis, microbes with significant differences in summer and winter water might cause the shift of geosmin; thus, the conditions that affect their growth should be illuminated. Redundant analysis (RDA), a PCA analysis constrained by environmental factors, could be employed to reveal the correlation of samples and environmental factors through a two-dimensional sequencing diagram (Figure 5). According to the RDA figure, temperature presents the highest correlation with the samples, which is negatively correlated with all other environmental factors, and distinguished the summer samples from winter samples clearly. It should be noticed that parameters other than temperature are in a sharp angle, suggesting they are in a positive correlation with each other, in terms of heavier eutrophication. The short distance between the summer water and sludge samples indicates that their composition is less affected by environmental parameters other than temperature, whereas the long distance between the winter samples imply that they are affected by other parameters besides temperature, especially for the winter sludge samples.

In view of the correlation between environmental factors and dominant microbes, hgcI clade, CL500-29 marine group, *Flavobacterium*, *Optitutae*, and *Sphingopyxis* are ranked as the top 5 genera positively correlated with the nutrients and geosmin, suggesting they might have a direct connection to the production or release of geosmin, especially in winter water. As two representative genera of Cyanobacteria, *Microcystis* is in negative correlation with the nutrient parameters and geosmin, and *Synechococcus* is almost in a right angle with geosmin, suggesting they might be nearly in no correlation with the geosmin. Moreover, the geosmin and nutrient parameters are nearly in the same direction, suggesting the positive correlation of nutrients to geosmin production, which is consistent with the previous works [33]. Therefore, regardless of what microbes are responsible for the production or release of geosmin, they must be in a positive correlation with the nutrient level; thus, removal of nutrients in raw water should be the primary measure against the geosmin pollution.

### 3.6. Co-Occurrence Correlations of Microorganisms and Environmental Parameters

**Figure 6:**
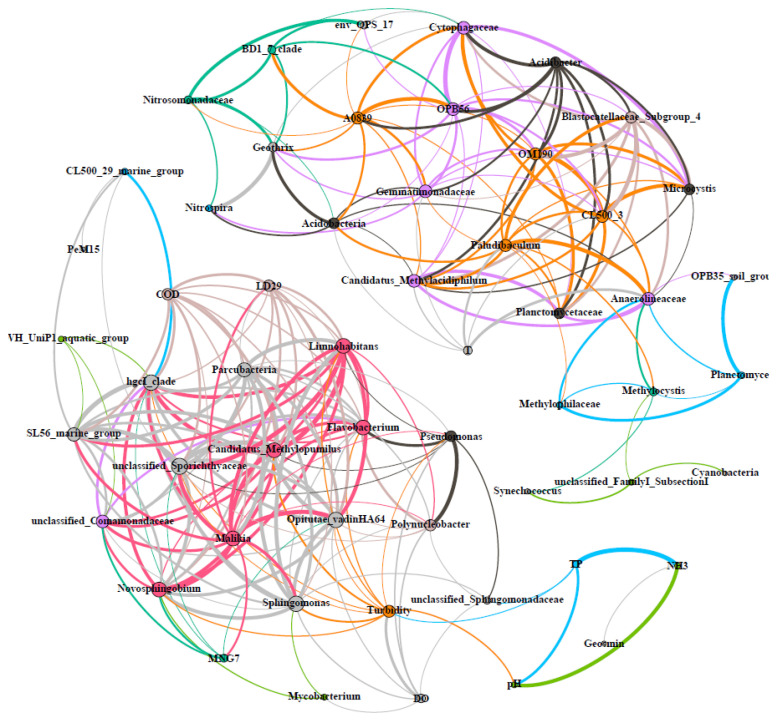
Network analysis of co-occurring bacterial genera and environmental parameters (the size of nodes is proportional to the number of connections, whereas the edges between two nodes are proportional to the value of Spearman’s correlation coefficients).

Comparing with the simple linear correlation revealed by RDA, network might be a more powerful tool in discovering the co-occurrence correlations among microbial communities and environmental parameters, verifying the complex inter-relationships between items with clean lines and nodes. According to the network analysis (with the Spearman’s coefficient > 0.60), the positive co-occurrence correlations among the top 50 genera and environmental parameters reveal that there are two distinct groups (Figure 6), in terms of the summer group being significantly correlated with temperature (upper-right) and the winter group being significantly correlated with COD, DO, and turbidity (bottom-left). The genera representing the freshwater plankton group are clustered together in the summer group, including *Planctomycetaceae*, *Cyanobacteria*, *Candidatus Methylacidiphilum*, *Synechococcus,* and *Microcystis*, which thrived in summer and withered in winter. The abundance ratios of these genera in summer and winter samples exceed 1.5, especially for the *Microcystis*, with a ratio of more than 100. According to the concentration of extracted DNA (Table S2), biomass in summer samples should be three times the winter samples; thus, the actual biomass of *Microcystis* in summer samples should be about 300 times that in winter samples. On the contrary, those genera representing the potential algae decomposers and geosmin degraders are clustered into the winter group, including the hgcI clade, CL500-29 marine group, *Flavobacterium, Methylopumilus,* and *Sphingopyxis*. These genera thrived in winter and withered in summer; thus, their abundance ratios in winter and summer samples are in the range of 1.2–25. It is noteworthy that the genera of *Novosphigobium*, *Sphigomonas,* and *Pseudomonas* could degrade geosmin efficiently when existing simultaneously along with geosmin [27]. In this study, all three genera were detected with a high abundance in winter sludge samples, being 10~100 times higher than that in summer water or sludge samples. Therefore, there might be a certain positive correlation between the abundance of these bacteria and the concentration of geosmin.

## 4. Conclusions

In this work, the concentration of geosmin in raw water was analyzed combining with the microbial population and sampling conditions, which reveals the release mechanism of geosmin in the field. Cyanobacteria bloomed in summer, whereas the concentration of geosmin increased sharply in winter along with the proliferation of Actinobacteria, but no specific OTUs in Actinobacteria could be assigned as the potential odor producer. Considering the properties of geosmin synthesis and storage of Cyanobacteria, as well as the microbial communities correlated with the decomposition of Cyanobacteria, geosmin might be produced and stored by Cyanobacteria in summer, which deposited into sediments after bloom and was decomposed by the heat-labile microbes such as hgcI clade, *Flavobacterium*, *Methylopumilus*, *Limnohabitans,* and *Sphingopyxis* in winter; thus, led the breakout of geosmin in raw water in winter.

## Data Availability

The sequencing raw reads were deposited into the NCBI Short-Reads Archive database with the accession numbers of PRJNA503301.

## Supplementary Materials

The following are available online at https://www.mdpi.com/article/10.3390/toxics10080485/s1, Figure S1: Phylogenetic tree of the top 50 OTUs and the reference sequences (blue, Actinobacteria; pink, Cyanobacteria), Table S1: Basic information of sampling date and water quality parameters; Table S2: Samples used for microbial community analysis and the sequencing data.

## References

[1] Gerber N.N., Lechevalier H.A. (1965). Geosmin, an earthly-smelling substance isolated from actinomycetes. Appl. Microbiol..

[2] Ma Z., Niu Y., Xie P., Chen J., Tao M., Deng X. (2013). Off-flavor compounds from decaying cyanobacterial blooms of Lake Taihu. J. Environ. Sci..

[3] Beniwal D., Taylor-Edmonds L., Armour J., Andrews R.C. (2018). Ozone/peroxide advanced oxidation in combination with biofiltration for taste and odour control and organics removal. Chemosphere.

[4] Antonopoulou M., Evgenidou E., Lambropoulou D., Konstantinou I. (2014). A review on advanced oxidation processes for the removal of taste and odor compounds from aqueous media. Water Res..

[5] Jüttner F., Watson S.B. (2007). Biochemical and ecological control of geosmin and 2-methylisoborneol in source waters. Appl. Environ. Microb..

[6] Su M., Suruzzaman M.D., Zhu Y.P., Lu J.P., Yu J.W., Zhang Y., Yang M. (2021). Ecological niche and in-situ control of MIB producers in source water. J. Envrion. Sci.-China.

[7] Olsen B.K., Chislock M.F., Wilson A.E. (2016). Eutrophication mediates a common off-flavor compound (2-methylisoborneol, in a drinking water reservoir. Water Res..

[8] Devi A., Chiu Y.T., Hsueh H.T., Lin T.F. (2021). Quantitative PCR based detection system for cyanobacterial geosmin/2-methylisoborneol (2-MIB) events in drinking water sources: Current status and challenges. Water Res..

[9] Mustapha S., Tijani J.O., Ndamitso M.M., Abdulkareem A.S., Shuaib D.T., Mohammed A.K. (2021). A critical review on geosmin and 2-methylisoborneol in water: Sources, effects, detection, and removal techniques. Environ. Monit. Assess.

[10] Zaitlin B., Watson S.B. (2006). Actinomycetes in relation to taste and odour in drinking water: Myths, tenets and truths. Water Res..

[11] Ma X., Feng J., Song Y., Ni M., Dietrich A.M., Chen C., Li Q., Gao N. (2015). Release behavior of odor contaminants derived from *Microcystis aeruginosa* in rivers and a non-strict anaerobic aqueous system. J. Water Supply Res. Technol..

[12] Clercin N.A., Druschel G.K., Gray M. (2021). Occurrences of 2-methylisoborneol and geosmin –degrading bacteria in a eutrophic reservoir and the role of cell-bound versus dissolved fractions. J. Envrion. Manag..

[13] Durrer M., Zimmermann U., Jüttner F. (1999). Dissolved and particle-bound geosmin in a mesotrophic lake (lake Zürich): Spatial and seasonal distribution and the effect of grazers. Water Res..

[14] Rice E., Baird R., Eaton A., Clesceri L. (2012). Standard Methods for the Examination of Water and Wastewater.

[15] Kozich J.J., Westcott S.L., Baxter N.T., Highlander S.K., Schloss P.D. (2013). Development of a dual-index sequencing strategy and curation pipeline for analyzing amplicon sequence data on the MiSeq Illumina sequencing platform. Appl. Environ. Microb..

[16] Liu L., Li H., Wang Z., Liu R., Zhang Y., Lin K. (2015). Insights into spatially and temporally co-occurring polybrominated diphenyl ethers in sediments of the East China Sea. Chemosphere.

[17] Jiang Y., Cheng B., Liu M., Nie Y. (2016). Spatial and temporal variations of taste and odor compounds in surface water, overlying water and sediment of the western Lake Chaohu, China. Bull. Environ. Contam. Toxicol..

[18] Wang Z., Li R. (2015). Effects of light and temperature on the odor production of 2-methylisoborneol-producing *Pseudanabaena sp.* and geosmin-producing *Anabaena ucrainica* (cyanobacteria). Biochem. Syst. Ecol..

[19] Zhang T., Lin L., Song L.R., Wei C. (2009). Effects of temperature and light on the growth and geosmin production of *Lyngbya kuetzingii* (Cyanophyta). J. Appl. Phycol..

[20] Li L., Yang S., Yu S., Zhang Y. (2019). Variation and removal of 2-MIB in full-scale treatment plants with source water from Lake Tai, China. Water Res..

[21] Sugiura N., Iwami N., Inamori Y., Nishimura O., Sudo R. (1998). Significance of attached cyanobacteria relevant to the occurrence of musty odor in Lake Kasumigaura. Water Res..

[22] Zeng D.N., Fan Z.Y., Chi L., Wang X., Qu W.D., Quan Z.X. (2013). Analysis of the bacterial communities associated with different drinking water treatment processes. World J. Microb. Biotechnol..

[23] Ávila M.P., Staehr P.A., Barbosa F.A.R., Chartone-Souza E., Nascimento A.M.A. (2017). Seasonality of freshwater bacterioplankton diversity in two tropical shallow lakes from the Brazilian Atlantic Forest. FEMS Microb. Ecol..

[24] Alghanmi H.A., Alkam F.M., AL-Taee M.M. (2018). Effect of light and temperature on new cyanobacteria producers for geosmin and 2-methylisoborneol. J. Appl. Phycol..

[25] Yuan R., Zhou B., Shi C., Yu L., Zhang C., Gu J. (2012). Biodegradation of 2-methylisoborneol by bacteria enriched from biological activated carbon. Front. Environ. Sci. Eng..

[26] Shimizu T., Oda T., Ito H., Imai I. (2017). Isolation and characterization of algicidal bacteria and its effect on a musty odor-producing cyanobacterium *Dolichospermum crassum* in a reservoir. Water Sci. Technol. Water Supply.

[27] Hoefel D., Ho L., Aunkofer W., Monis P., Keegan A., Newcombe G., Saint C. (2006). Cooperative biodegradation of geosmin by a consortium comprising three gram-negative bacteria isolated from the biofilm of a sand filter column. Lett. Appl. Microb..

[28] Ho L., Hoefel D., Bock F., Saint C.P., Newcombe G. (2007). Biodegradation rates of 2-methylisoborneol (MIB) and geosmin through sand filters and in bioreactors. Chemosphere.

[29] Salcher M.M., Pernthaler J., Zeder M., Psenner R., Posch T. (2008). Spatio-temporal niche separation of planktonic Betaproteobacteria in an oligo-mesotrophic lake. Environ. Microbiol..

[30] Salcher M.M., Neuenschwander S.M., Posch T., Pernthaler J. (2015). The ecology of pelagic freshwater methylotrophs assessed by a high-resolution monitoring and isolation campaign. ISME J..

[31] Šimek K., Kasalický V., Jezbera J., Horňák K., Nedoma J., Hahn M.W., Bass D., Jost S., Boenigk J. (2013). Differential freshwater flagellate community response to bacterial food quality with a focus on Limnohabitans bacteria. ISME J..

[32] Paver S.F., Hayek K.R., Gano K.A., Fagen J.R., Brown C.T., Davis-Richardson A.G., Crabb D.B., Rosario-Passapera R., Giongo A., Triplett E.W. (2013). Interactions between specific phytoplankton and bacteria affect lake bacterial community succession. Environ. Microbiol..

[33] Oh H.S., Lee C.S., Srivastava A., Oh H.M., Ahn C.Y. (2017). Effects of environmental factors on Cyanobacterial production of odorous compounds: Geosmin and 2-Methylisoborneol. J. Microbiol. Biotechnol..

