# Supplementary material for: Insights into the Seasonal Olfactory Mechanism of Geosmin in Raw Water of Huangpu River
Source: Toxics. 2022 Aug 19;10(8):485. doi: 10.3390/toxics10080485 (PMC9415234; doi:10.3390/toxics10080485)
Supplement: Supplementary file 1 [file toxics-10-00485-s001.zip › toxics-1846274-supplementary.pdf]

toxics MDPI

Supplementary Materials: Insights into the Seasonal Olfactory Mechanism of Geosmin in Raw Water of Huangpu River

Fei Luo, Hui Chen, Xiaoxin Wu, Lili Liu, Yuean Chen and Zhiping Wang

Table S1. Basic information of sampling date and water quality parameters.

Sample No.	Date	Temperature		Weather	Wind	Turbidity (NTU)	DO	COD	NH_3	TP	Geosmin
		High	Low				(mg/L)				(ng/L)
1	2016-03-21 Monday	15°C	10°C	Cloudy~ Overcast	East wind 1-2 class	18	6.42	3.98	1.52	0.09	6.48
2	2016-04-01 Friday	22°C	13°C	Sunny~ Cloudy	Southwest wind 1-2 class	21	6.58	4.65	1.64	0.08	8.87
3	2016-04-14 Thursday	25°C	15°C	Cloudy	Southwest wind 1-2 class	20	6.31	4.47	1.87	0.08	4.60
4	2016-04-26 Tuesday	19°C	15°C	Little ~ Moderate rain	East wind 1-2 class	35	6.35	4.51	1.85	0.07	<0.20
5	2016-05-12 Thursday	28°C	19°C	Sunny	Southwest wind 1-2 class	27	5.81	4.16	1.61	0.07	11.14
6	2016-05-26 Thursday	26°C	20°C	Moderate rain	North wind 1-2 class	47	4.57	3.82	1.19	0.06	11.50
7	2016-06-01 Wednesday	21°C	18°C	Moderate rain	Northeast wind 4-5 class	42	4.98	3.66	1.1	0.06	<0.20
8	2016-06-13 Monday	28°C	21°C	Cloudy	Northeast wind 1-2 class	28	4.76	4.18	1.36	0.07	5.82
9	2016-06-29 Wednesday	27°C	23°C	Shower	South wind 1-2 class	32	4.53	4.57	1.58	0.07	3.22
10	2016-07-08 Friday	31°C	26°C	Shower	East wind 1-2 class	31	4.63	5.29	1.34	0.06	4.78
11	2016-07-21 Thursday	38°C	30°C	Sunny	Southwest wind 1-2 class	24	4.31	5.07	1.34	0.06	6.03
12	2016-07-29 Friday	38 °C	29 °C	Thunder shower~ Cloudy	South wind 1-2 class	25	4.57	5.31	1.46	0.06	7.57
13	2016-08-12 Friday	34 °C	28 °C	Cloudy	Southeast wind 1-2 class	27	4.24	5.22	1.32	0.08	5.72
14	2016-08-19 Friday	36 °C	29 °C	Sunny	Southeast wind 1-2 class	22	4.19	5.58	1.29	0.08	6.23
15	2016-09-02 Friday	35 °C	26 °C	Cloudy	West wind 1-2 class	26	4.26	5.54	1.4	0.06	5.5
16	2016-09-09 Friday	31 °C	24 °C	Cloudy~ Overcast	Northeast wind 1-2 class	28	4.63	5.26	1.28	0.06	4.88
17	2016-09-26 Monday	30 °C	24 °C	Rain showers	East wind 1-2 class	32	4.77	5.14	0.98	0.06	4.21
18	2016-10-10 Monday	22 °C	18 °C	Cloudy~ Overcast	Northeast wind 1-2 class	26	5.34	4.32	0.69	0.07	<0.20
19	2016-10-24 Monday	23 °C	20 °C	Overcast	Southeast wind 1-2 class	27	5.59	4.27	0.76	0.07	<0.20

20	2016-11-07 Monday	23 °C	14 °C	Little ~ Moderate rain	Southeast wind 1-2 class	38	5.73	4.15	0.83	0.08	<0.20
21	2016-11-29	13 °C	10 °C	Overcast Little rain	Southeast wind 1-2 class	35	5.51	4.92	1.32	0.09	20.34
22	2016-12-05 Monday	18 °C	9 °C	Cloudy~ Overcast	Southwest wind 1-2 class	34	5.86	5.74	1.99	0.11	20.86
23	2016-12-12 Monday	16 °C	11 °C	Cloudy~ Little rain	Southwest wind 1-2 class	33	6.56	6.28	2.54	0.13	19.46
24	2016-12-19 Monday	18 °C	13 °C	Cloudy~ Little rain	East wind 1-2 class	31	6.29	6.53	2.29	0.12	12.71

Rows marked with blue color are used to represent summer samples, while rows marked with gray color represented the winter samples.

Table S2. Samples used for microbial community analysis and the sequencing data.

Sequncing No.	Sampling date & Type	Nucleic acid (ng/μL)	Absorbance 260/280	Sequences	Coverage	Chao index	Shannon index
11#	2016-07-21 Sludge	53.3	1.79	43813	0.97	5266.736	6.601554
12#	2016-08-12 Sludge	78.1	1.78	34084	0.97	4551.797	6.423157
13#	2016-09-02 Sludge	82.5	1.85	46676	0.97	5033.161	6.385886
14#	2016-09-09 Sludge	86.6	1.80	39470	0.96	5495.265	6.481221
15#	2016-09-26 Sludge	108.5	1.79	38934	0.96	5222.335	6.331765
16#	2016-07-21 Sludge	48.6	1.91	48942	0.97	5098.838	6.581536
17#	2016-08-12 Sludge	94.9	1.85	44160	0.97	4864.628	6.32521
18#	2016-09-02 Sludge	70.6	1.79	48445	0.97	5679.119	6.656059
19#	2016-09-09 Sludge	183.5	1.80	40688	0.96	5992.327	6.498348
20#	2016-09-26 Sludge	39.4	1.80	33880	0.97	4292.571	6.360061
21#	2016-07-21 Water	54.3	1.76	52374	0.98	4896.708	6.457025
22#	2016-08-12 Water	48.1	1.76	43663	0.97	5128.583	6.212478
23#	2016-09-02 Water	88.2	1.85	50173	0.97	5433.633	6.36689
24#	2016-09-09 Water	36.9	1.83	60663	0.98	5468.484	6.431954
25#	2016-09-26 Water	84	1.83	47523	0.97	5353.144	6.231605
26#	2016-12-12 Sludge	32.5	1.86	53076	0.99	2148.800	4.280681
27#	2016-12-19 Sludge	18.1	1.95	47960	0.98	3706.402	5.606855
28#	2016-12-12 Water	23.1	1.89	61724	0.99	2750.442	4.487307
29#	2016-12-19 Water	31.7	1.86	49745	0.99	2443.396	4.571827

Rows marked with blue color are used to represent summer samples, while rows marked with gray color represented the winter samples.

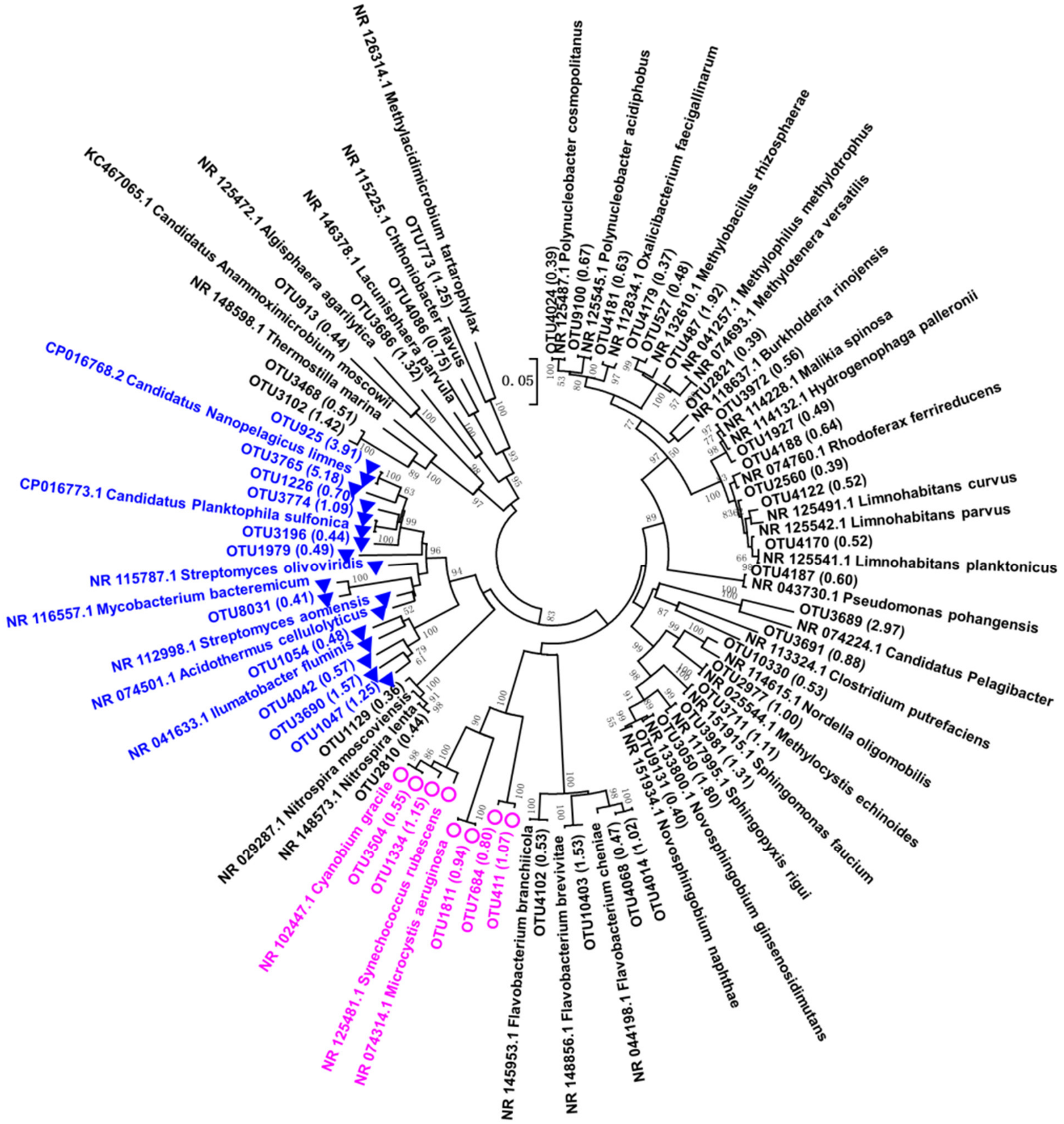

Figure S1. Phylogenetic tree of top 50 OTUs and the reference sequences (Blue, Actinobacteria; Pink, Cyanobacteria).
